# Supplementary material for: Senescence drives immunotherapy resistance by inducing an immunosuppressive tumor microenvironment
Source: Nat Commun. 2024 Mar 18;15:2435. doi: 10.1038/s41467-024-46769-9 (PMC10948808; doi:10.1038/s41467-024-46769-9)
Supplement: Supplementary file 1 — Supplementary Information [file 41467_2024_46769_MOESM1_ESM.pdf]

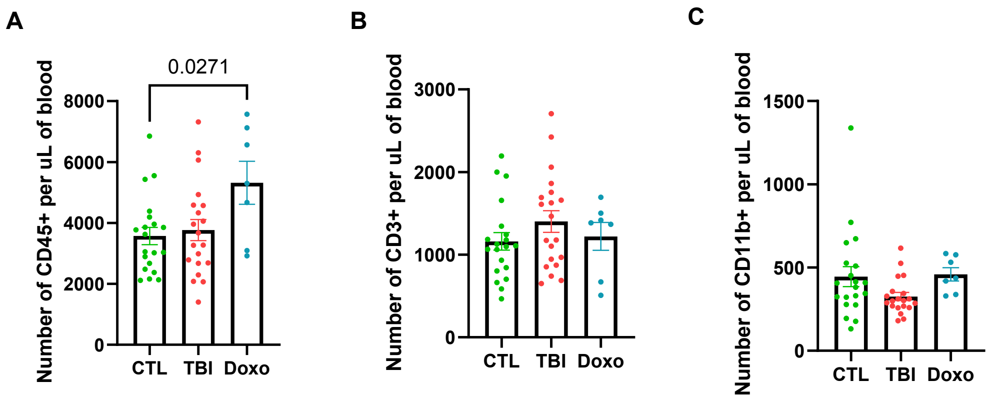

Supplementary Fig 1: Hematopoietic cell count is not affected long-term by senescence.
The absolute number of circulating CD45+ (panel A), CD3+ (panel B) and CD11b+ (panel C) cells in blood was evaluated by flow-cytometry in control mice and 12 or 4 weeks after mice were subjected to TBI or doxorubicin (Doxo) respectively. n = 20 CTL, n=20 TBI and n=7 Doxo mice. Ordinary one way ANOVA with Tukey correction. Shown is the Mean±SEM. Source data are provided as a Source Data file.

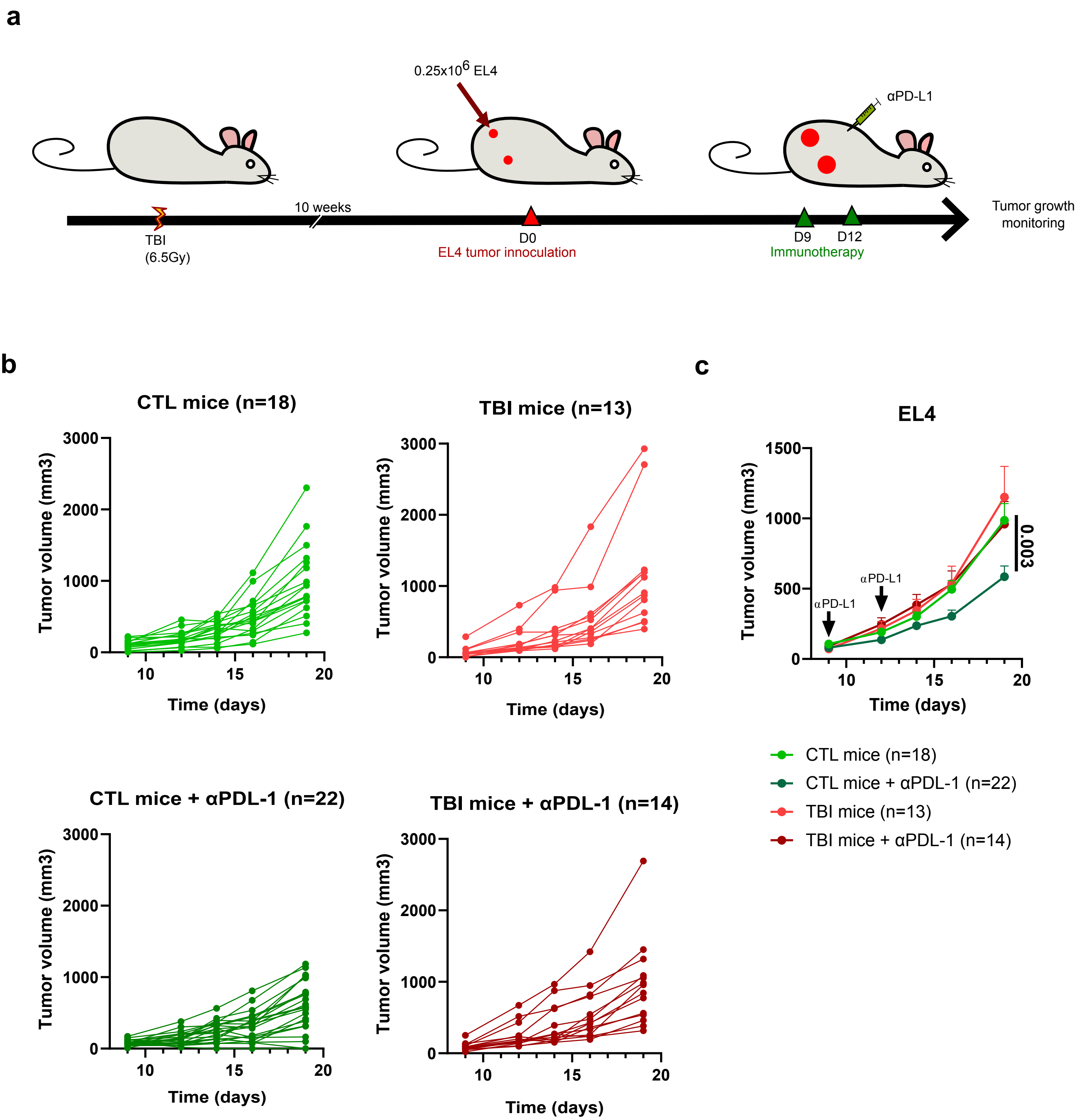

Supplementary Fig. 2: Resistance of EL-4 tumors to αPD-L1 immunotherapy in TBI mice.
A) Schematic of the experimentation. TIS was induced by TBI and 10 weeks later 2.5x105 EL-4 tumor cells were injected subcutaneously in the right and left flanks. Mice were then injected intraperitoneal with a blocking αPD-L1 antibody on days 9 and 12 post tumor inoculation.
B) Tumor growth was evaluated for each of the indicated groups of mice. Each line represents the growth of each individual tumors over 20 days or until the mouse had to be removed from the study. The total number of tumors per group is indicated in parenthesis.
C) Shown is the mean size of each individual tumors. 2-way ANOVA with Tukey correction. The total number of tumors per group is indicated in parenthesis. Source data are provided as a Source Data file.

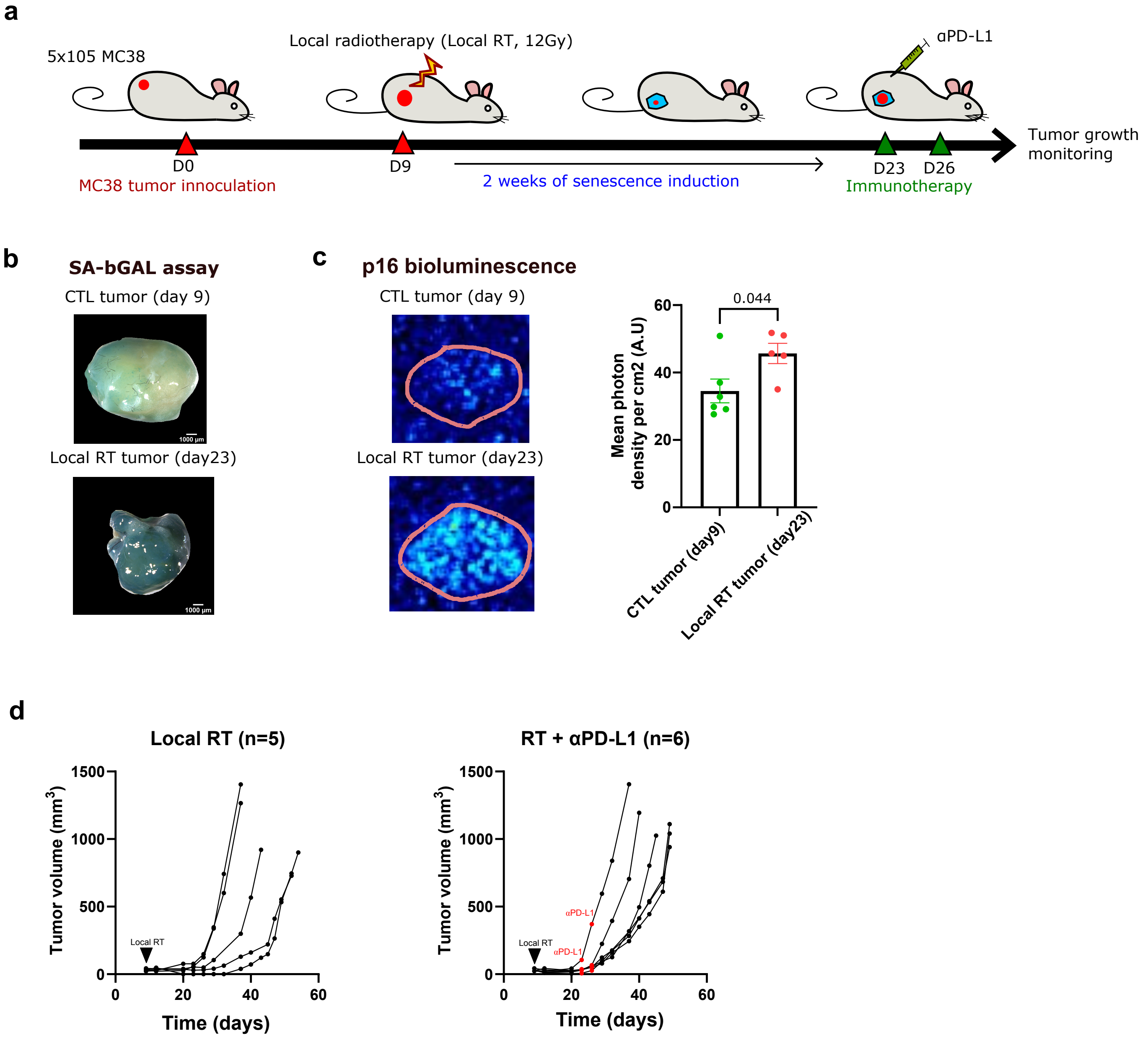

Supplementary Fig 3: Locally induced senescence decreases the efficacy of immunotherapy
A) Schematic of the experimentation. Established MC38 tumors (approximately 50mm3 in size – day 9 post inoculation) were exposed to a single 12 Gy dose of local radiotherapy (RT) to induce senescence. 14 days after RT, tumors were either excised to measure senescence induction or mice were treated with an αPD-L1 antibody.
B) Representative images of a control tumor (day 9) and a tumor exposed to RT (day 23) stained for SA-βGal activity.
C) Representative images and quantification of p16 bioluminescence in a control tumor (day 9) and a tumor 23 days after exposure to RT. N = 5-6 per group. Two-tailed unpaired T-test. Shown is the Mean±SEM.
D) Individual tumor growth was measured in mice subjected or not to the combination of local RT and αPD-L1 treatments. Number of mice for each group is showed in parenthesis. Source data are provided as a Source Data file.

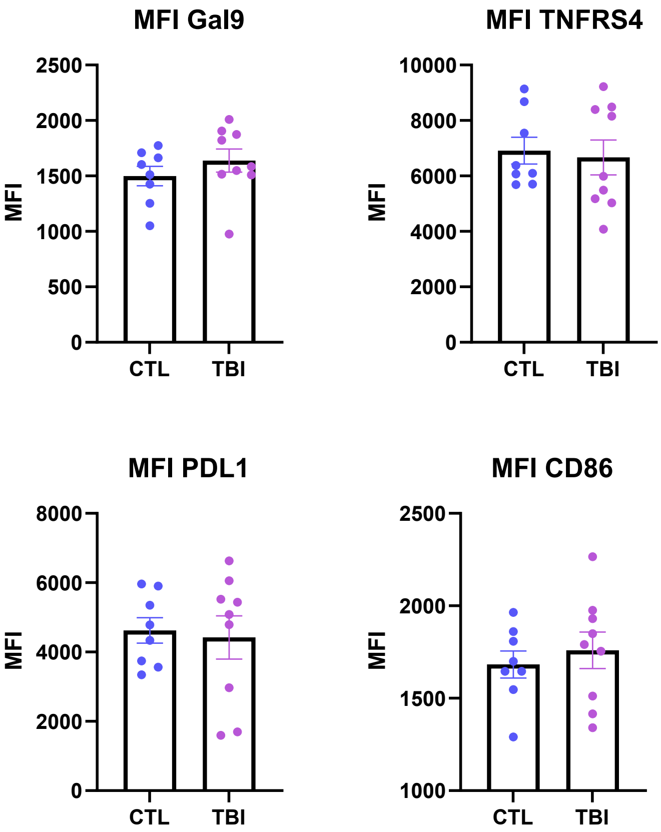

Supplementary Fig 4: Expression of inhibitory ligands on MC38 tumor cells.
MC38 tumors were collected from control (CTL) and TBI mice 20 days post inoculation, dissociated and the expression of inhibitory ligands measured by flow cytometry. Shown is the mean fluorescence intensity (MFI) per tumor cells. n=8 CTL and n= 9 TBI tumors from independant animals. Unpaired two tailed T test. Shown is the mean +/- SEM. Source data are provided as a Source Data file.

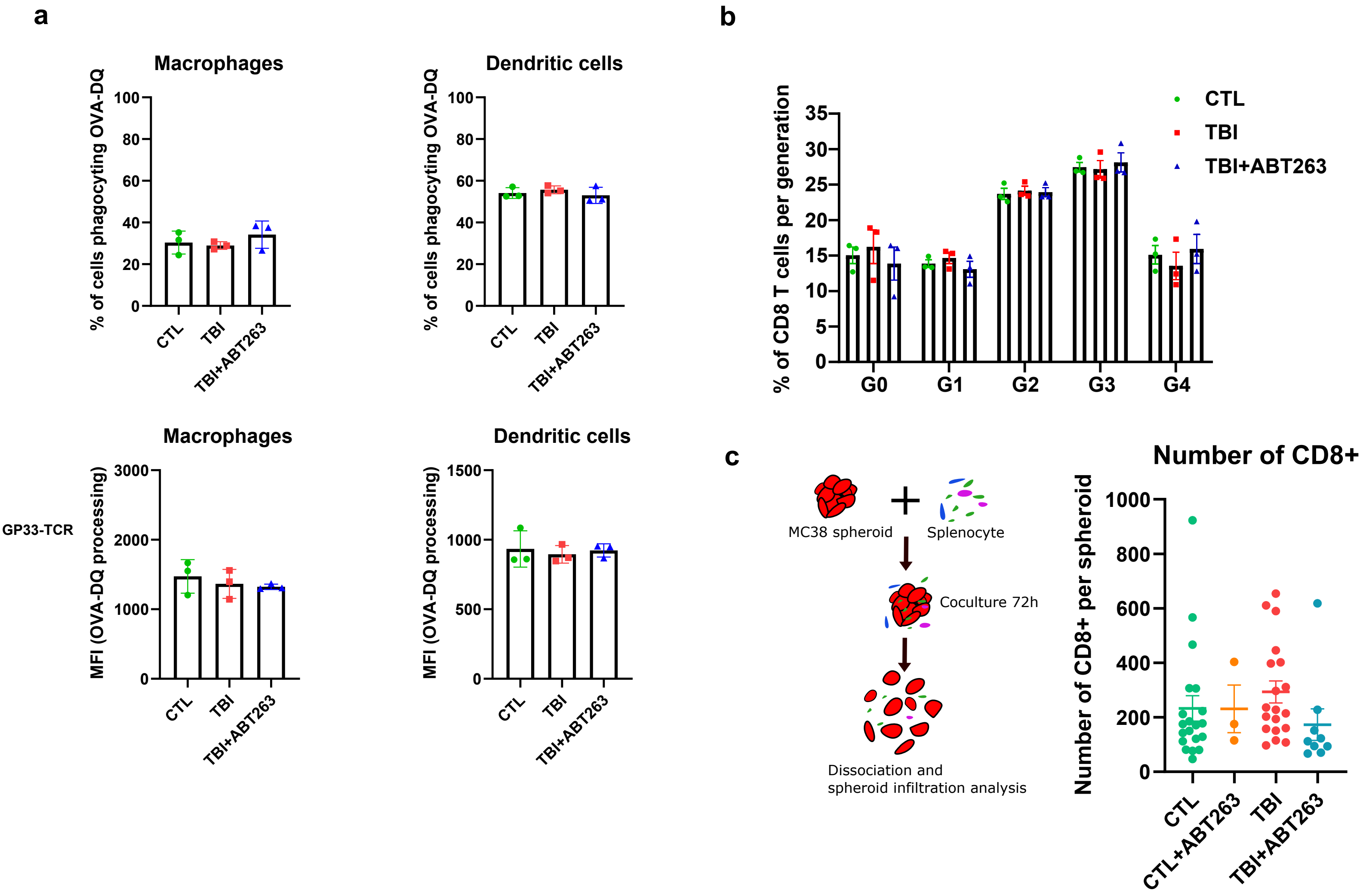

Supplementary Fig 5: The infiltration and antigen presentation capacity of splenocytes collected from TBI mice is not impaired.

A) Splenocytes were collected from the indicated groups of mice and the capacity of macrophages and dendritic cells to phagocyte the OVA-DQ recombinant protein was evaluated by flow cytometry. Shown is the percentage of cells able to phagocyte OVA-DQ and the mean +/- SEM fluorescence intensity of the digested fragments of DQ ovalbumin after intracellular processing. n=3 splenocytes isolated from independant animals. Ordinary ONE way ANOVA with Tukey correction.

B) Splenocytes were collected from the indicated groups of mice and exposed to the gp33 peptide for 12h to allow cross presentation. CD8 T cells collected from P14 mice were labelled with CFSE and then added to the gp33 pulsed splenocytes. CD8 T cell proliferation was evaluated by flow cytometry 72 hours later. 2way ANOVA with tukey correction. n=3 CD8 T cells isolated from independant spleens. Shown is the mean +/- SEM.

C) MC38 spheroids were formed in vitro and co-culture with splenocytes collected from the indicated groups of mice. Spheroids were washed, enzymatically dissociated and immune cell infiltration evaluated by flow cytometry 72 hours later. Ordinary ONE way ANOVA with Tukey correction. n=20 CTL, n=4 CTL+ABT263, n=19 TBI and n=9 TBI+ABT263 splenocytes isolated from independant animals. Shown is the mean +/- SEM. Source data are provided as a Source Data file.

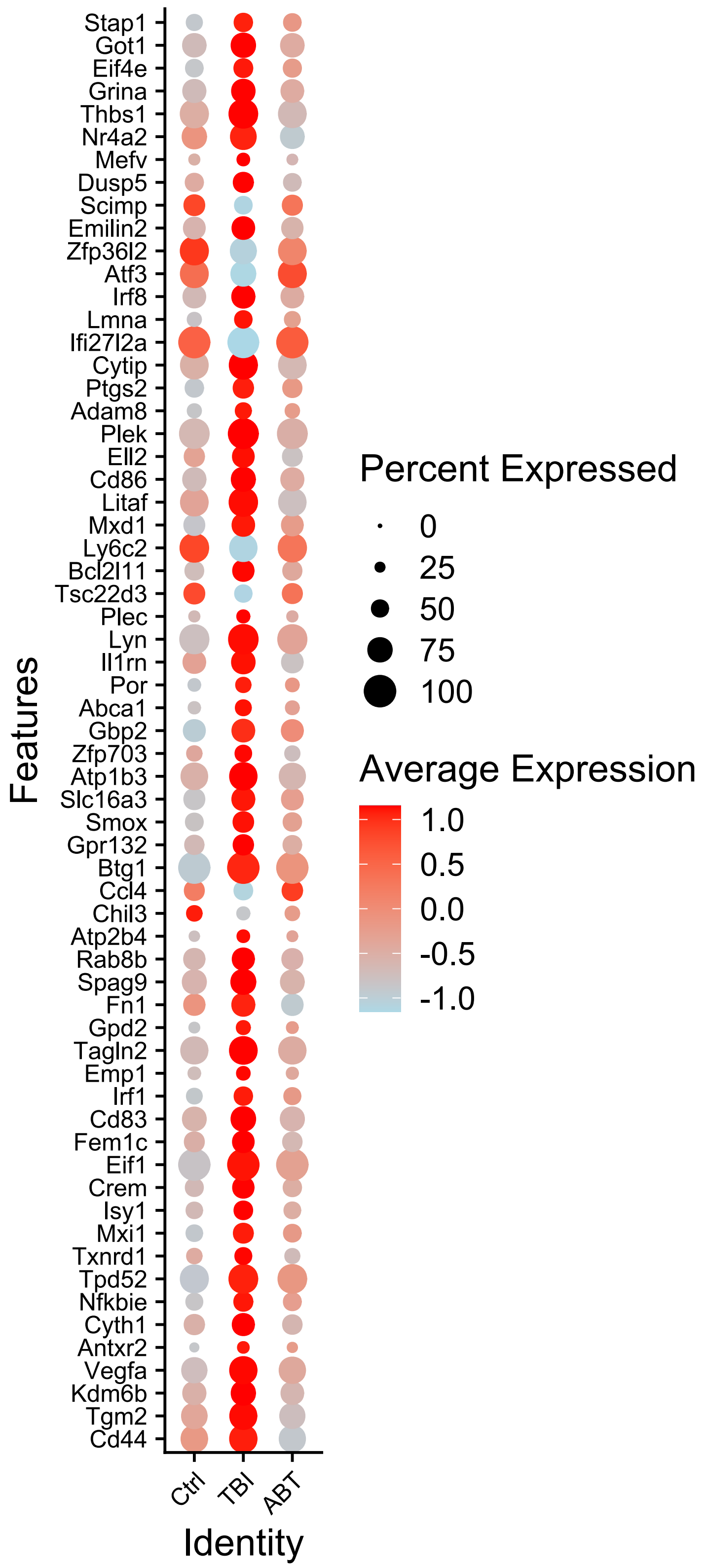

Supplementary Fig 6: Complete list of the 63 DEGs restored after ABT263 treatment in TBI monocytes.
DotPlot of the 63 DEGs restored after ABT263 treatment in TBI isolated monocytes cluster.

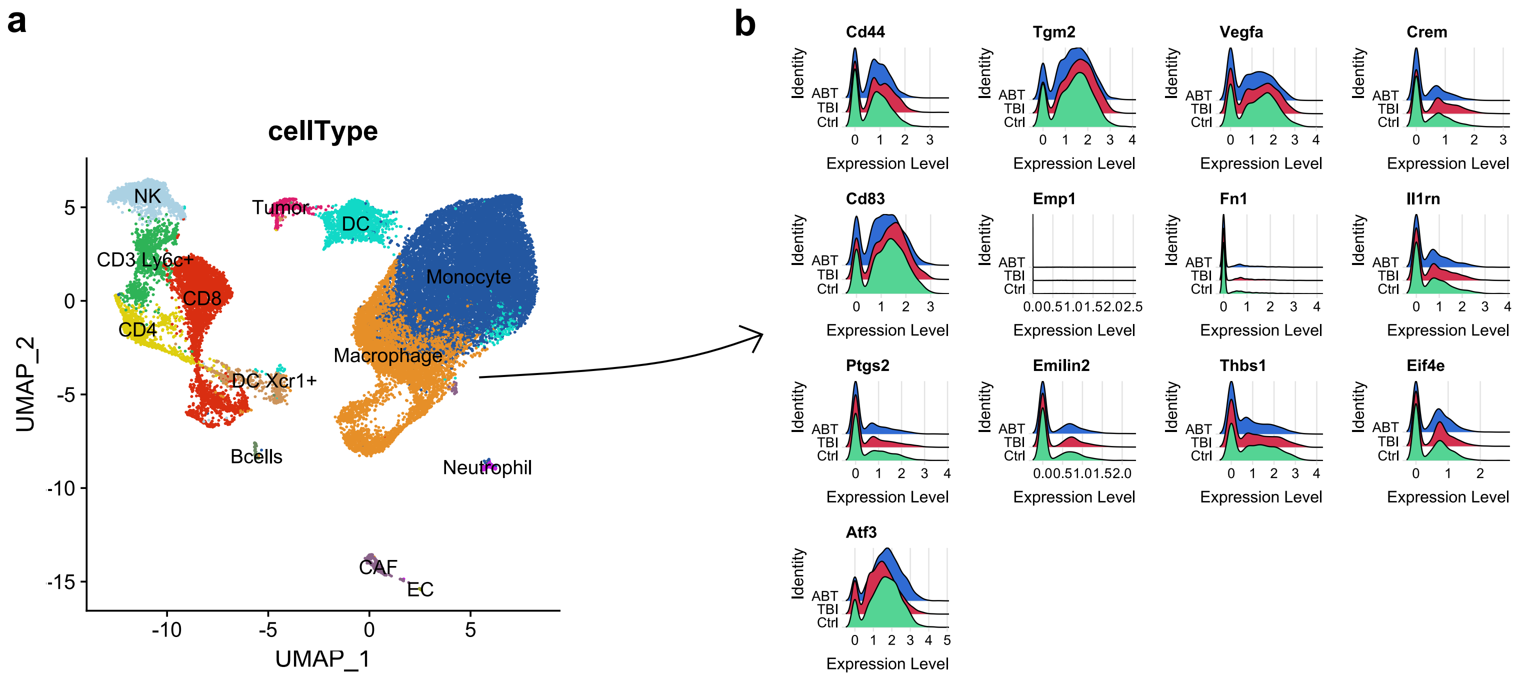

Supplementary Fig 7 : The immunosuppressive transcriptomic signature in macrophages collected from TBI mice.

A) The transcriptomic signature of macrophages collected from tumors of the indicated groups of mice was analyzed using the TBI-Monocyte-Signature generated in figure 5. n=1634 macrophages for Ctrl, n=2133 macrophages for TBI, n=2566 macrophages for ABT.

B) Shown is the ViolinPlot representation for the gene expression of each indicated genes.

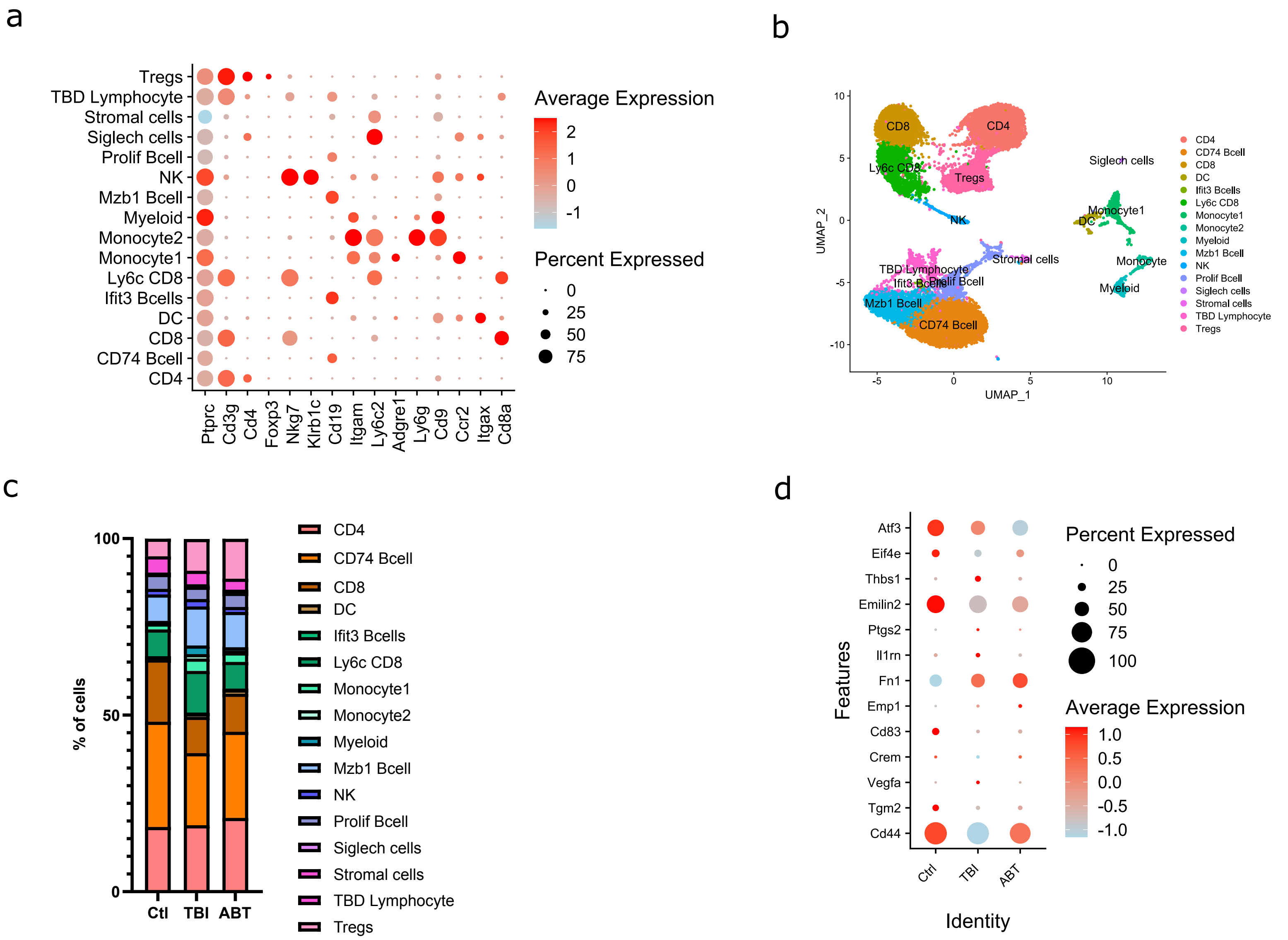

Supplementary Fig 8: The immunosuppressive transcriptomic signature is partially found in splenocytes collected from tumor free TBI mice.

A) Splenocytes were dissociated from tumor free TBI mice and used to perform a single-cell transcriptomic analysis. n=5131 cells for CTL, n=7000 cells for TBI and n= 5604 cells for ABT. Shown is the DotPlot representation of genes used to identify cell clusters in the spleen.

B) UMAP representation of cell clusters composing the spleen.

C) Shown is the percentage of each clusters of the different groups of mice.

D) Shown is a DotPlot representation of the TBI-Monocyte-Signature in monocytes (Itgam+, Ly6c2+).

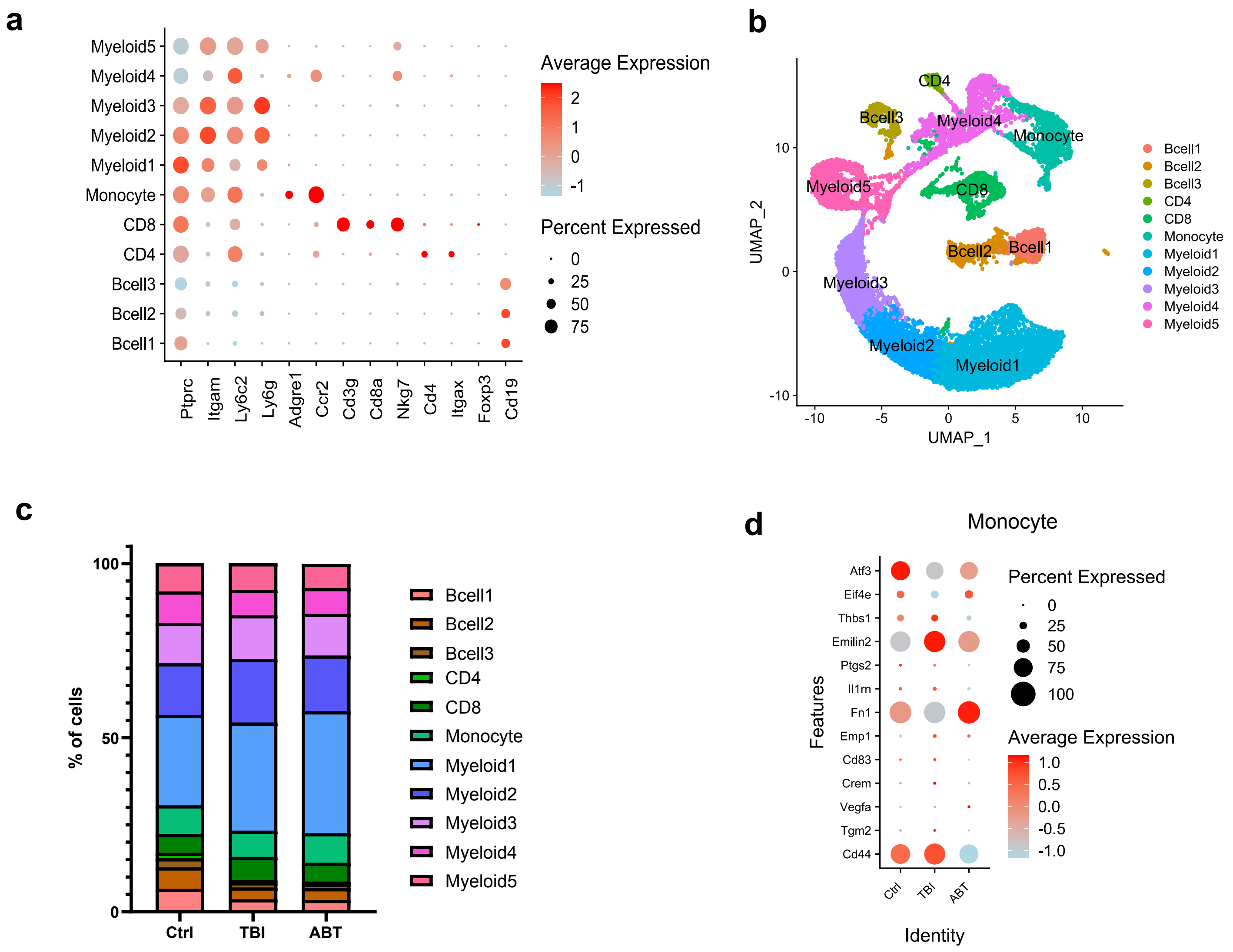

Supplementary Fig. 9: The immunosuppressive transcriptomic signature is partially found in bone marrow cells collected from tumor free TBI mice.

A) BM cells were dissociated from tumor free TBI mice and used to perform a single cell transcriptomic analysis. n=6181 cells for CTL, n=6373 cells for TBI and n=7053 cells for ABT. Shown is the DotPlot representation for genes used to identify cells clusters in the BM.

B) UMAP representation of cell clusters composing the BM.

C) Shown is the percentage of each clusters of the different group of mice.

D) Shown is a DotPlot representation of the TBI-Monocyte-Signature in monocytes (Itgam+, Ly6c2+, Ccr2+).

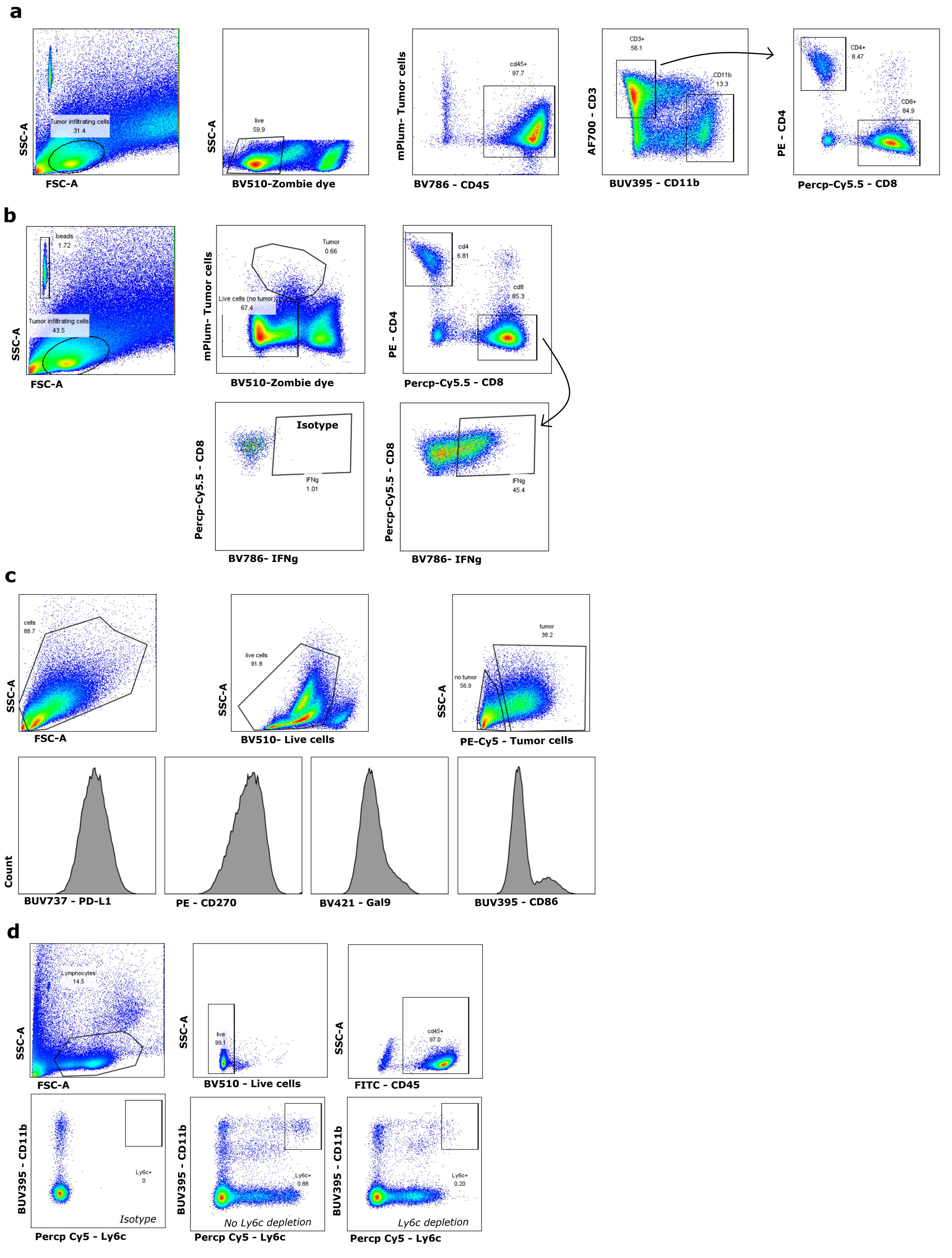

Supplementary Fig. 10: gating strategy for Flow-cytometry
a. Gating strategy for tumor infiltrating cell
b. Gating strategy for IFNg expression in CD8
c. Gating strategy for TCIR
d. Gating strategy for blood Ly6c depletion
