# Supplementary material for: Senescence drives immunotherapy resistance by inducing an immunosuppressive tumor microenvironment
Source: Nat Commun. 2024 Mar 18;15:2435. doi: 10.1038/s41467-024-46769-9 (PMC10948808; doi:10.1038/s41467-024-46769-9)
Supplement: Supplementary file 3 — Description of Additional Supplementary Files [file 41467_2024_46769_MOESM3_ESM.pdf]

Description of Additional Supplementary Files

Supplementary Data 1: complete list of DEGs defining clusters.

Supplementary Data 2: complete list of DEGs identified after using FindMarkers function between Ctrl, TBI and ABT group in the different clusters.

Supplementary Data 3: Antibodies used for flow cytometry.
