# Supplementary material for: Senescence drives immunotherapy resistance by inducing an immunosuppressive tumor microenvironment
Source: Nat Commun. 2024 Mar 18;15:2435. doi: 10.1038/s41467-024-46769-9 (PMC10948808; doi:10.1038/s41467-024-46769-9)
Supplement: Supplementary file 7 — Reporting Summary [file 41467_2024_46769_MOESM7_ESM.pdf]

nature portfolio

Corresponding author(s): Christian Beauséjour

Last updated by author(s): Jan 25, 2024

Reporting Summary

Nature Portfolio wishes to improve the reproducibility of the work that we publish. This form provides structure for consistency and transparency in reporting. For further information on Nature Portfolio policies, see our Editorial Policies and the Editorial Policy Checklist.

Statistics

For all statistical analyses, confirm that the following items are present in the figure legend, table legend, main text, or Methods section.

n/a	Confirmed	
☐	☒	The exact sample size (*n*) for each experimental group/condition, given as a discrete number and unit of measurement
☐	☒	A statement on whether measurements were taken from distinct samples or whether the same sample was measured repeatedly
☐	☒	The statistical test(s) used AND whether they are one- or two-sided<br>*Only common tests should be described solely by name; describe more complex techniques in the Methods section.*
☐	☒	A description of all covariates tested
☐	☒	A description of any assumptions or corrections, such as tests of normality and adjustment for multiple comparisons
☐	☒	A full description of the statistical parameters including central tendency (e.g. means) or other basic estimates (e.g. regression coefficient) AND variation (e.g. standard deviation) or associated estimates of uncertainty (e.g. confidence intervals)
☐	☒	For null hypothesis testing, the test statistic (e.g. *F*, *t*, *r*) with confidence intervals, effect sizes, degrees of freedom and *P* value noted<br>*Give P values as exact values whenever suitable.*
☒	☐	For Bayesian analysis, information on the choice of priors and Markov chain Monte Carlo settings
☒	☐	For hierarchical and complex designs, identification of the appropriate level for tests and full reporting of outcomes
☒	☐	Estimates of effect sizes (e.g. Cohen's *d*, Pearson's *r*), indicating how they were calculated

Our web collection on statistics for biologists contains articles on many of the points above.

Software and code

Policy information about availability of computer code

Data collection	BD FACSDiva, LabeoTech In vivo imaging System
Data analysis	Cell Ranger v6.1.2, R package Seurat v4.0, NovaSeq S6000, DoubletFinder v2.0.2, fgsea package ver1.28, ImageJ software v1.53, GraphPad Prism v9.1.0

For manuscripts utilizing custom algorithms or software that are central to the research but not yet described in published literature, software must be made available to editors and reviewers. We strongly encourage code deposition in a community repository (e.g. GitHub). See the Nature Portfolio guidelines for submitting code & software for further information.

Data

Policy information about availability of data

All manuscripts must include a data availability statement. This statement should provide the following information, where applicable:

- Accession codes, unique identifiers, or web links for publicly available datasets
- A description of any restrictions on data availability
- For clinical datasets or third party data, please ensure that the statement adheres to our policy

The single cell transcriptomic data generated in this study have been deposited in the GEO database under accession code GSE256486 [https://www.ncbi.nlm.nih.gov/geo/query/acc.cgi?acc=GSE256486].

Research involving human participants, their data, or biological material

Policy information about studies with human participants or human data. See also policy information about sex, gender (identity/presentation), and sexual orientation and race, ethnicity and racism.

Reporting on sex and gender	N/A
Reporting on race, ethnicity, or other socially relevant groupings	N/A
Population characteristics	N/A
Recruitment	N/A
Ethics oversight	N/A

Note that full information on the approval of the study protocol must also be provided in the manuscript.

Field-specific reporting

Please select the one below that is the best fit for your research. If you are not sure, read the appropriate sections before making your selection.

[x] Life sciences [ ] Behavioural & social sciences [ ] Ecological, evolutionary & environmental sciences

For a reference copy of the document with all sections, see nature.com/documents/nr-reporting-summary-flat.pdf

Life sciences study design

All studies must disclose on these points even when the disclosure is negative.

Sample size	sample size were measured using power calculation excel sheet (https://www.bu.edu/research/ethics-compliance/animal-subjects/animal-care/research/sample-size-calculations-iacuc/) as determined when we submitted our project for animal ethics approval.
Data exclusions	Mice were rejected when tumor did not grow or when tumor growth was in asynchrony for treatment.
Replication	Our results were validated using two approaches (a genetic approach using GCV and a pharmacological approach using ABT263). Each in vivo experiments involving tumor growth were repeated at least 2 time with a minimun of 3 mice per groups. In vitro experiment were repeated at least 3 times with similar results.
Randomization	mice distribution was random
Blinding	Investigators were not blind as tumors size were not determined using unbiased live cell imaging. Similarly, immune cell counts were obtained using flow cytometry on the entire dissociated tissues leaving no place for interpretation of the data.

Reporting for specific materials, systems and methods

We require information from authors about some types of materials, experimental systems and methods used in many studies. Here, indicate whether each material, system or method listed is relevant to your study. If you are not sure if a list item applies to your research, read the appropriate section before selecting a response.

Materials & experimental systems

n/a	Involved in the study	
[ ]	[x]	Antibodies
[x]	[ ]	Eukaryotic cell lines
[x]	[ ]	Palaeontology and archaeology
[ ]	[x]	Animals and other organisms
[x]	[ ]	Clinical data
[x]	[ ]	Dual use research of concern
[x]	[ ]	Plants

Methods

n/a	Involved in the study	
[x]	[ ]	ChIP-seq
[ ]	[x]	Flow cytometry
[x]	[ ]	MRI-based neuroimaging

Antibodies

Antibodies used	Antibodies for flow cytometry:<br>CD45-BV785: Brilliant Violet 785™ anti-mouse CD45 Antibody, Clone 30-F11, Cat# 103149, Biolegend. Dilution 1/100

CD3-AF700: Alexa Fluor® 700 anti-mouse CD3 Antibody, Clone 17A2, Cat# 100216, Biolegend. Dilution 1/100
CD4-PE: PE anti-mouse CD4 Antibody, Clone H129.19, Cat#130310, Biolegend. Dilution 1/100
CD8-PercpCy5: PerCP/Cyanine5.5 anti-mouse CD8a Antibody, Clone 53-6.7, Cat# 100734, Biolegend. Dilution 1/100
CD11b-BUV395: BD Horizon™ BUV395 Rat Anti-CD11b, Clone M1/70, Cat#563553, BDBioscience. Dilution 1/100
IFNG-BV785: Brilliant Violet 785™ anti-mouse IFN-γ Antibody, Clone XMG1.2, Cat#505837, Biolegend. Dilution 1/100
CD11c-APC: APC anti-mouse CD11c Antibody, clone N418, Cat#117310, Biolegend. Dilution 1/100
F4/80-APC-Cy7: APC/Cyanine7 anti-mouse F4/80 Antibody, Clone BM8, Cat# 123117, Biolegend. Dilution 1/100
CD45-PE-Cy7: PE/Cyanine7 anti-mouse CD45 Antibody, Clone 30-F11, Cat#103113, Biolegend. Dilution 1/100
CD8-BV421: Brilliant Violet 421™ anti-mouse CD8a Antibody, Clone 53-6.7, Cat#100737, Biolegend. Dilution 1/100
BUV737 Rat Anti-Mouse CD274 : BD OptiBuild™ BUV737 Rat Anti-Mouse CD274, clone MIH5. Dilution 1/100
BV421 Rat Anti-Mouse Galectin-9: BD Horizon™ BV421 Rat Anti-Mouse Galectin-9, clone RG9-35. Dilution 1/100
PE anti-mouse CD270 (HVEM) Antibody: Clone HMHV-1B18, Biolegend, dilution 1/100
BD Horizon™ BUV395 Rat Anti-Mouse CD86: Clone GL1, dilution1/100
FITC-CD45: FITC anti-mouse CD45 Antibody, clone 30-F11, Cat#103107, Biolegend, dilution 1/100

In vivo treatment:
anti Ly6c: InVivoMAb anti-mouse Ly6C, Clone Monts1, Cat# BE0203, BioXcell
anti PD-L1: InVivoMAb anti-mouse PD-L1 (B7-H1), Clone 10F.9G2, Cat#BE0101, BioXcell
anti CTTLA4: InVivoMAb anti-mouse CTLA-4 (CD152), Clone 9H10, Cat#BE0131, BioXcell

Validation

Each antibodies were previously validated by the manufacturer (Biolegend, BD Biosciences and BioXcell). For flow cytometry, all antibodies were also validated in our cell population by a comparison with control isotype.
In vitro:
CD45-BV785: Mouse reactivity verified by manufacturer. Podd BS, et al. 2006. J. Immunol. 176:6532. (FC, CMCD) PubMed
CD3-AF700: Mouse reactivity verified by manufacturer. Hirai T, et al. 2020. Immunity. 54(1):84-98.e5. PubMed
CD4-PE: Mouse reactivity verified by manufacturer. Lawson H, et al. 2021. Stem Cell Reports. 16:2784. PubMed
CD8-PercpCy5: Mouse reactivity verified by manufacturer. Shih FF, et al. 2006. J. Immunol. 176:3438. (FC)
CD11b-BUV395: Mouse reactivity verified by manufacturer. Driver DJ, McHeyzer-Williams LJ, Cool M, Stetson DB, McHeyzer-Williams MG. Development and maintenance of a B220- memory B cell compartment. J Immunol. 2001; 167(3):1393-1405. (Clone-specific: Flow cytometry, Immunofluorescence.
IFNG-BV785: Mouse reactivity verified by manufacturer. Ferrick D, et al. 1995. Nature 373:255. (FC)
CD11c-APC: Mouse reactivity verified by manufacturer.Ramakrishna C, et al. 2019. Nat Commun. 10:2153. PubMed
F4/80-APC-Cy7:Mouse reactivity verified by manufacturer. Rosina M, et al. 2022. Cell Metab. 34:533. PubMed
CD45-PE-Cy7: Mouse reactivity verified by manufacturer. Tran NT, et al. 2019. Cell Rep. 28:3510. PubMed
CD8-BV421: Mouse reactivity verified by manufacturer.Jayachandran R, et al. 2019. Immunity. 50:152. PubMed
BUV737 Rat Anti-Mouse CD274: Ansari MJ, Salama AD, Chitnis T, et al. The programmed death-1 (PD-1) pathway regulates autoimmune diabetes in nonobese diabetic (NOD) mice. J Exp Med. 2003 July; 198(1):63-69. (Biology).
BV421 Rat Anti-Mouse Galectin-9: Chiba S, Baghdadi M, Akiba H, et al. Tumor-infiltrating DCs suppress nucleic acid-mediated innate immune responses through interactions between the receptor TIM-3 and the alarmin HMGB1.. Nat Immunol. 2012; 13(9):832-42. (Clone-specific: In vivo exacerbation)
PE anti-mouse CD270 (HVEM) Antibody:Verma S, et al. 2016. J Virol. 90: 650 - 658.
BD Horizon™ BUV395 Rat Anti-Mouse CD86: Bluestone JA. New perspectives of CD28-B7-mediated T cell costimulation. Immunity. 1995; 2(6):555-559.
CD45-FITC: Podd BS, et al. 2006. J. Immunol. 176:6532. (FC, CMCD) PubMed

In vivo:
anti Ly6c: Rowe, A. M., et al. (2017). "Subclinical Herpes Simplex Virus Type 1 Infections Provide Site-Specific Resistance to an Unrelated Pathogen" J Immunol. doi : 10.4049/jimmunol.1601310.
anti PD-L1: Grasselly, C., et al. (2018). "The Antitumor Activity of Combinations of Cytotoxic Chemotherapy and Immune Checkpoint Inhibitors Is Model-Dependent" Front Immunol 9: 2100.
anti CTTLA4: Ariyan, C. E., et al. (2018). "Robust Antitumor Responses Result from Local Chemotherapy and CTLA-4 Blockade" Cancer Immunol Res 6(2): 189-200.

Animals and other research organisms

Policy information about studies involving animals; ARRIVE guidelines recommended for reporting animal research, and Sex and Gender in Research

Laboratory animals

12-14 weeks old p16-3MR mice on C57bl/6 background were used. Mice were housed in the animal care facility at the CHU Sainte-Justine Research Center under pathogen-free conditions in sterile ventilated racks. Mice were housed on a 12:12 h light:dark cycle at 21 °C and 40% humidity, and they had free access to food (Harlan Teklad 2918) and tap water.

Wild animals

This study did not involve wild animals.

Reporting on sex	both male and female mice were used in the study. No difference between sex was observed when sufficent number of mice were available for statistical analysis.
Field-collected samples	This study did not involve samples collected from the field.
Ethics oversight	Comité Institutionnel des Bonnes Pratiques Animales en Recherche of the CHU Ste-Justine.

Note that full information on the approval of the study protocol must also be provided in the manuscript.

Plants

Seed stocks	*Report on the source of all seed stocks or other plant material used. If applicable, state the seed stock centre and catalogue number. If plant specimens were collected from the field, describe the collection location, date and sampling procedures.*
Novel plant genotypes	*Describe the methods by which all novel plant genotypes were produced. This includes those generated by transgenic approaches, gene editing, chemical/radiation-based mutagenesis and hybridization. For transgenic lines, describe the transformation method, the number of independent lines analyzed and the generation upon which experiments were performed. For gene-edited lines, describe the editor used, the endogenous sequence targeted for editing, the targeting guide RNA sequence (if applicable) and how the editor was applied.*
Authentication	*Describe any authentication procedures for each seed stock used or novel genotype generated. Describe any experiments used to assess the effect of a mutation and, where applicable, how potential secondary effects (e.g. second site T-DNA insertions, mosiacism, off-target gene editing) were examined.*

Flow Cytometry

Plots

Confirm that:

☒ The axis labels state the marker and fluorochrome used (e.g. CD4-FITC).

☒ The axis scales are clearly visible. Include numbers along axes only for bottom left plot of group (a 'group' is an analysis of identical markers).

☒ All plots are contour plots with outliers or pseudocolor plots.

☒ A numerical value for number of cells or percentage (with statistics) is provided.

Methodology

Sample preparation	Immune cells were obtained after tumor dissociation (Miltenyi tumor dissociation kit, cat#130.096.730).
Instrument	LSR Fortessa flow cytometer
Software	FACS-diva for acquisition and FlowJo v10 for data analysis
Cell population abundance	Post tumor dissociation, more than 1 000 000 cells were analysed. Approximatively 50% of analysed cells are live immune cells (15% lymphoid cells, 35% myeloid cells, 5% others).
Gating strategy	For TME immune cells experiments:<br>FSC/SSC gates were used to select cells of interest and exclude debris. Doublets were eliminated by using FSC-W/FSC.-H and SSC-W/SSC-H gates. Tumor cells were removed by selecting negative mPlum cells in SSC-A/APC-Cy5 gates. Live cells were selected by using SSC-A/BV510-Acqua-zombi dye gates. Immune CD45+ cells selected by using positive cells in SSC-A/CD45-BV785 gates. Lymphoid and myeloid cells were separated by using CD3-AF700/CD11b-BUV395 gates. CD8 and CD4 cells were discriminated by using CD4-PE/CD8-percyCy5 staining.<br><br>IFNg evaluation:<br>Doublets, tumor cells and dead cells were eliminated as previoulsy described. Lymphoid cells are first isolated by using SSC-A/CD3-AF700 gates. CD8 and CD4 cells were discriminated by using CD4-PE/CD8-percyCy5 gates. IFNg positive cells were measured by using CD4-PE/IFNg-BV785 or Percp.Cy5-CD8/IFNg-BV785 gates.

☒ Tick this box to confirm that a figure exemplifying the gating strategy is provided in the Supplementary Information.
